# The Sophisticated Transcriptional Response Governed by Transposable Elements in Human Health and Disease

**DOI:** 10.3390/ijms21093201

**Published:** 2020-04-30

**Authors:** Federica Marasca, Erica Gasparotto, Benedetto Polimeni, Rebecca Vadalà, Valeria Ranzani, Beatrice Bodega

**Affiliations:** 1 Fondazione INGM, Istituto Nazionale di Genetica Molecolare “Enrica e Romeo Invernizzi”, 20122 Milan, Italy; marasca@ingm.org (F.M.); gasparotto@ingm.org (E.G.); polimeni@ingm.org (B.P.); vadala@ingm.org (R.V.); ranzani@ingm.org (V.R.); 2 Translational and Molecular Medicine, DIMET, University of Milan-Bicocca, 20900 Monza, Italy

**Keywords:** transposable elements, co-option, genome plasticity, immune system response, cancer progression, next generation sequencing approaches

## Abstract

Transposable elements (TEs), which cover ~45% of the human genome, although firstly considered as “selfish” DNA, are nowadays recognized as driving forces in eukaryotic genome evolution. This capability resides in generating a plethora of sophisticated RNA regulatory networks that influence the cell type specific transcriptome in health and disease. Indeed, TEs are transcribed and their RNAs mediate multi-layered transcriptional regulatory functions in cellular identity establishment, but also in the regulation of cellular plasticity and adaptability to environmental cues, as occurs in the immune response. Moreover, TEs transcriptional deregulation also evolved to promote pathogenesis, as in autoimmune and inflammatory diseases and cancers. Importantly, many of these findings have been achieved through the employment of Next Generation Sequencing (NGS) technologies and bioinformatic tools that are in continuous improvement to overcome the limitations of analyzing TEs sequences. However, they are highly homologous, and their annotation is still ambiguous. Here, we will review some of the most recent findings, questions and improvements to study at high resolution this intriguing portion of the human genome in health and diseases, opening the scenario to novel therapeutic opportunities.

## 1. Introduction

### 1.1. Transposable Elements (TEs) Account for Genome Evolution and Inter-Individual Genetic Variability

Two thirds of the human genome are composed of repetitive elements (66%), among which transposable elements (TEs) alone account for the 40–45% of human genome composition [1,2]. One fascinating question for genome biologists is to untangle the functions of this “dark side” of the genome, that still represents “alive matter” which evolution can influence to generate novel functions. It is clear nowadays that TEs capability of regulating the genome resides mainly in generating a sophisticated plethora of RNA regulatory networks, which in turn influence the transcriptional output of the cell [3,4,5]. TEs are organized into four different classes and, with the exception of DNA transposons, are mainly retrotransposons, which have acquired the ability by using RNA as intermediate to move via a ‘copy and paste’ mechanism. Retrotransposons include long interspersed elements (LINEs), short interspersed elements (SINEs), and long terminal repeat (LTR) retrotransposons. They are further classified as autonomous or non-autonomous depending on whether they have open reading frames (ORFs) that encode for the machinery required for the retrotransposition [6].

**Figure 1:**
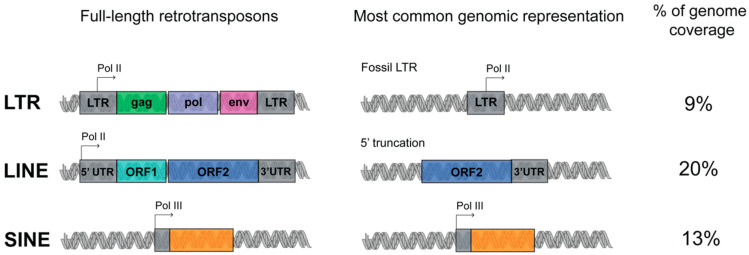
Schematic representation of retrotransposons classes organization. Retrotransposons are divided in three major classes: long interspersed elements (LINE), short interspersed elements (SINE) and long terminal repeat (LTR). Left, full length retrotransposons: the regulatory sequences are represented in grey; RNA Pol II and Pol III promoters are indicated with arrows; the protein coding sequences are indicated with colors. Middle, most common transposable elements (TEs) in the human genome. Right, retrotransposon coverage of the human genome (see the main text for details).

LINE is a class of transposon that is very ancient and evolutionary successful. Three LINE superfamilies are found in the human genome, namely LINE1, LINE2 and LINE3, of which only LINE1 is still active. Full-length LINE1 (L1) elements are approximately 6 kb long and constitute an autonomous component of the genome. A LINE1 element has an internal polymerase II promoter and encodes for two open reading frames, ORF1 and ORF2 (Figure 1) [7]. Once the L1 RNA is transcribed, it is exported to the cytoplasm for translation, and subsequently assembled with the chaperone RNA- binding proteins ORF1 and the endonuclease and reverse transcriptase ORF2. These ribonucleoparticles are then reimported into the nucleus, where ORF2 makes a single-stranded nick and primes reverse transcription from the 3′ end of the L1 RNA. Reverse transcription frequently results in many truncated, nonfunctional insertions, and for this reason, most of the LINE-derived repeats are short, with an average size around 900–1000 bp. The L1s are estimated to be present in more than 500,000 copies in the human genome [7].

The L1 machinery is also responsible for the retrotransposition of the SINEs (which can be classified into three superfamilies: Alu, MIR, MIR3), non-autonomous retroelements without any coding potential, short in length (around 300 bp) and transcribed from polymerase III promoter (Figure 1). The most represented human specific SINE superfamily, the Alu, is represented in 1,090,000 copies in the human genome [8].

The LTR retrotransposons are initiated and terminated by long terminal direct repeats embedded by transcriptional regulatory elements. The autonomous LTR retrotransposons contain *gag* and *pol* genes, which encode a reverse transcriptase, integrase, protease and RNAse H (Figure 1). Four superfamilies of LTR exist: ERV- class I, ERV(K) class II, ERV(L) class III, and MalR. MalR is the most represented superfamily of LTR, present in 240,000 copies [9].

Evolutionary biologists hypothesize that self-replicating RNA genomes were the basis of early life on earth, and that the advent of reverse transcription had a pivotal function in the evolution of the first DNA genomes, the more stable deoxyribose-based polymers [6,10]. From this perspective, multiple rounds of reverse transcription could have helped to expand both the size and complexity of the human genome. It is particularly evident in both mammals and plants that retrotransposons have massively accumulated, driving genome evolution. It is reported that L1 and Alu represent the most prominent catalysts of the human genome evolution [11] and that homologous recombination between TEs could have driven/drives mutations, chromosome rearrangement, deletions, inversions and translocations [12]. TEs are a major source of somatic genomic diversity and interindividual variability [13] and TE insertions have been documented as physiological occurrences [14,15,16]. In particular L1 retrotransposition has been extensively described as taking place in neurons, from fly to man [17,18,19], a mechanism that is fine-tuned and epigenetically regulated in neural progenitor development and differentiation, contributing to the somatic diversification of neurons in the brain [13,20]. The deregulation of TEs activity is nowadays emerging as an important contributor to many different diseases, as it occurs in neurological and inflammatory diseases and cancers [21,22,23].

The hosts have developed many systems to control TEs expression and expansion [24] (thus, epigenetic modification and noncoding RNAs (ncRNA) such Piwi interacting-RNAs) to contain the possible detrimental effects of their retrotransposition. This expansion has achieved a balance between detrimental and beneficial effects, possibly becoming a novel regulatory mechanism to promote genomic functions acquired through evolution [3]. It is nowadays accepted, both in mouse and in human, that TEs have been co-opted into multiple regulatory functions for the accommodation of the host genomes metabolisms and transcription, mediated both by their DNA elements and by their transcribed RNAs counterparts.

### 1.2. Not Just Transposition: TEs RNAs Are a Prolific Source for Novel Regulatory Functions

TEs were first discovered in maize by Barbara McClintock almost 80 years ago. She suggested these elements as “controlling elements” able to regulate the genes activity [25,26]. Her theories, even if dismissed for a long time, were pioneering and with the advent of next generation sequencing (NGS) technologies have been thoroughly revised. Currently emerging is the concept that TEs interact with the transcriptional regulatory functions of the hosts genomes [3,4,27,28].

Although a massive portion of the literature has been centered on the study of the retrotransposition and the effects of the de novo insertions, it is worth noting that TEs can have RNA regulatory functions decoupled from their retrotransposition.

International decade-long projects such as ENCODE (Encyclopedia of DNA Elements) and FANTOM (Functional Annotation of the Mammalian Genome) have produced and bioinformatically analyzed a vast number of datasets opening the way for studying TEs. These results revealed that TEs have precise functions in establishing and influencing the cell type specific transcriptional programs, creating regulatory networks that are fostered both by their genomic elements and the derived transcripts [3,28], revealing that the RNAs transcribed from this elements could have a myriad of functions, definitely changing the way in which many genomic concepts were written in textbooks [29].

These studies clarified that TEs can create novel or alternative promoters [30], promote the assembly of transcription factors [31] and epigenetic modifiers and favor their spreading [32] and the regulation of gene expression. Further, TEs in particular SINEs and HERVs, have been demonstrated to have functions in 3D genome folding, as the binding sites for chromatin organizers [33,34,35].

In the 2009 Faulkner et al. [36], demonstrated for the first time that TEs are widely expressed in human and mouse cell types with tissue-specific patterns of expression, suggesting a specific spatiotemporal activation of retrotransposons. Faulkner et al. further demonstrated that up to the 30% of the transcripts initiate within repetitive elements [36]. It is interesting to notice that tissues of embryonic origin contain the highest proportion of transposable element-derived sequences in their transcriptomes, with specific expression of LTR in placenta and oocytes [37]. In accordance, it was recently found that different classes of repeats are specifically enriched in genes with a definite spatiotemporal expression, further dictating their timing and magnitude of expression in development [38].

Within this scenario, TEs magnify the transcriptome complexity in different ways: generating antisense transcripts, usually in proximity to gene promoters [36], acting on the maturation of mRNAs via nursing alternative splicing sites for tissue specific exonization [39,40], and providing alternative polyadenylation signals [41,42] and sites for the RNA-mediated decoy [43]. Furthermore, TEs contribute to RNA regulatory sequences within introns and untranslated regions (UTRs) [36]. It is important to notice that TEs are major contributors to long noncoding RNAs (lncRNAs) [44,45]. In this scenario, an enhancer RNAs function was proposed for LTR derived transcripts, as required for pluripotency maintenance in mouse and human embryonic stem (ES) cells [46,47]. Further, it has been demonstrated that LINEs and SINEs are expressed as RNAs tightly associated to the chromatin compartment, where they localized at euchromatin, suggesting a possible function of these RNAs in 3D genome folding [48]. L1s have been described also as chromatin associated RNAs both in embryogenesis, regulating open chromatin accessibility [49,50], and in mouse ES cells, where they are involved in the regulation of genes required for cell identity maintenance and two-cell stage differentiation [51].

Although these seminal papers have increased the awareness and knowledge of the functions of TEs, highlighting important epigenetic roles for transposons in embryogenesis and development, the contribution of TEs to adult cell plasticity and diseases occurrence and progression is still poorly investigated. This is a result of the intrinsic difficulties in studying TEs, which due to their repetitive nature, high degree of homology, sequence divergence, and degeneration, render almost unfeasible the application of the technologies established for biallelic genes, in particular in bioinformatic.

Here, we will revise the TEs mediated multi-faced functions in promoting the establishment of a sophisticated plethora of RNA regulatory networks, which in turn influence the transcriptional plasticity of the cells. We will show how TEs transcriptional deregulation in pathological context is instead instrumental to fuel diseases. In particular we will review how TEs RNA can become a key player in the regulation of the immune response, using cell intrinsic specific pathways to directly control the regulation of interferon production and the activation of the immune cells; the alteration of these phenomena occurs in autoimmune and inflammatory diseases. Similarly, transcriptional deregulation of TEs represents a hallmark of cells that have lost identity, such as in cancer cells, where TEs onco-co-optation represents an important way to evolve cancer specific functions to promote tumor fitness and survival. Many of these findings have been achieved through the employment of NGS technologies with the application of bioinformatic pipelines that are in continuous evolution. Within this frame, an unambiguous TE identification and expression quantification of TEs at the genomic instance level would allow the precise and systematic definition of their contribution to RNA regulatory networks. We will review advances in the field and the challenges that should be addressed in this direction.

## 2. The Transcriptional Role of TEs in Shaping the Innate and Adaptive Immune Response

### 2.1. TEs RNAs Boost Innate and Adaptive Immune Response

**Figure 2:**
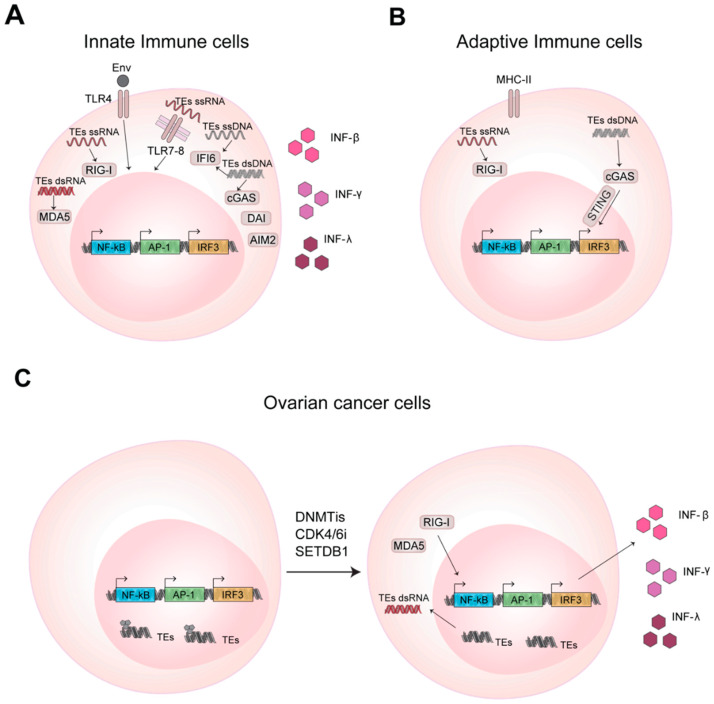
TEs promote innate and adaptive immune response activation in health and disease through RNA and DNA sensing pathways. (**A**) Nucleic acids of TEs bind and activate the transmembrane Toll-like receptors (TLRs) and cytosolic pattern recognition receptors (PRRs) activating transcription factors that promotes *INF* gene transcription and IFNs production. (**B**) TEs in T and B lymphocytes activate adaptive immune response through RNA and DNA sensing pathways, as mentioned in (**A**). (**C**) In cancer cells the inhibition of DNA methylation, promotes TEs expression and enhances cytokines production.

The immune system is able to protect our organism against pathogens and foreign substances thanks to an innate and adaptive immune response [52]. During evolution, TEs have established transcriptional networks acting as regulatory DNA elements and also as signaling molecules for the immune system. The RNAs transcribed from TEs and/or the corresponding reverse transcribed cDNA are used by the host sensing pathways to promote expression of interferons that further solicits the immune response [53,54,55] (Figure 2A).

Alu can act as regulatory elements for *IFN-β* genes providing the binding site for the key transcription factor NF-κB [56]. Similarly, Thomson et al., [57] in the 2009 discovered that *IFN-γ* locus is highly enriched for TEs, where a cluster of ERVL-Alu Sx-ERVL is required for the NF-κB dependent activation of *IFN-γ* expression in response to LPS [57]. Recently, Choung et al. [58] demonstrated that elements originating from LTR work as IFN-inducible enhancers, a function conserved among different mammalian species, suggesting an evolutive co-option of TEs elements to regulate the expression of genes related to immunity [58].

A viral infection can promote the immune response through different mechanism, that include not only the recognition of the viral capsid protein or surface glycoprotein of the viruses, but also involve the sensing pathways for cytosolic foreign RNA and DNA, both single- and double-stranded (i.e., ssRNA, dsRNA, ssDNA, dsDNA) [59]. These molecules activate the immune response binding the host pattern recognition receptors (PRRs), that are transmembrane receptors as Toll-like receptors (TLRs) or the cytosolic receptors as RIG-I and MDA5. ERVs can promote the antiviral immune response through the encoded env protein, the transcribed RNA or reverse transcribed cDNA that are recognized by the host PRRs receptors [60]. In 2006, Rolland et al. [61] found that the envelope of HERV-W, a member of specific superfamily of ERV elements, binds the TLR4 located on the cellular membrane, inducing the secretion of proinflammatory cytokines and the stimulation of monocytes and dendritic cells that in turns promote the CD4^+^ T effector cells response [61] (Figure 2A). In 2004 Heil et al. [62] demonstrated that other isoforms of TLR, as TLR7 and TLR8, localized in the endosomal membranes, are able to bind cytosolic HERV ssRNA. Cytosolic PRRs are very sensitive in detecting cytosolic RNA and Chiappinelli et al. [63] in 2015 demonstrated that HERV dsRNA binds MDA5, promoting IFN-β production (Figure 2A). However, in analogy to ERVs, other TEs can also regulate the activation of the immune response, stimulating the same pathways. L1 RNA, due to its AU-rich sequence, can be-recognized by RIG-I and MDA5 [64]. Cytosolic L1 RNA is recognized by RIG-I through its 5′ terminal triphosphate form (a feature common to TEs) and L1 dsRNA binds MDA5. These interactions promote *IFN-β* expression [65]. Similarly, also the cytosolic dsRNA derived from Alu could induce the transcription of inflammatory genes and inhibit viral protein synthesis [66,67].

Besides the RNA-sensing pathways, TEs are able to elicit the immune response also through DNA-sensing pathways, often stimulated by their cytosolic, reverse transcribed ssDNA or cDNA [60,68,69,70] (Figure 2A). In B cells, it has been demonstrated that ERVs could promote cell activation and the production of antigen-specific antibodies through both their cytosolic RNAs and cDNAs, the former activating RIG-I-MAVS and the latter cGAS-STING (GMP-AMP synthase (cGAS), adaptor stimulator of IFN genes (STING)) pathways [71] (Figure 2B). In the same context, L1 retrotransposition is instead inhibited by the cytidine deaminase AID (activation-induced cytidine deaminase) that, by reducing ORF1 protein level, promotes a strict surveillance of retrotransposon accumulation in the cytoplasm. Importantly, mutation in AID promotes the increase of cytosolic L1 RNA and cDNA, contributing to the autoimmune phenotype typical of diseases that show defects in the *AID* gene as hyper-IgM syndrome [72].

Overall, we provide evidence that TEs, taking advantage of the machinery used by the viral genomes, are intriguing novel players able to fine tune and regulate the immune response, shaping TEs as possible novel targets for immunological approaches.

### 2.2. Deregulation of the Expression Levels of TEs is Implicated in Autoimmunity and Inflammation

Several studies have demonstrated the function of TEs in innate and adaptive immune response via IFN regulation with multiple mechanisms. This concept envisages the deregulation of TEs as a possible key component in the development of inflammation and autoimmune diseases.

In 2016, Manghera et al. [73] demonstrated that ERVK overexpression in amyotrophic lateral sclerosis (ALS) could represent a connection between the neuronal damage in ALS and the impaired signaling by proinflammatory cytokines signaling. In ALS, it has been demonstrated that ERVK reactivation occurs in the neurons of the motor cortex in ALS. ERVK promoter retains two conserved interferon-stimulated response elements (ISREs) that are activated in the motor neuron by the proinflammatory cytokines signaling typical of ASL. ERVK expression in turn contributes to a neurodegenerative phenotype nursing the inflammatory response using the above-mentioned sensing pathways, identifying ERVK as novel players of the pathology [73,74]. Similarly, another study reported that ERVK expression or the env protein translation cause retraction and beading of neurites in human neurons. A mice transgenic model expressing the ERVK env indeed developed motor dysfunction and a loss of volume in the motor cortex, impaired synaptic activity in pyramidal neurons, defects in the dendritic spine and DNA damage increase [75]. These studies define that ERVK reactivation can contribute to degeneration of motor neuron, possibly via different mechanisms, identifying ERVK as novel biomarker of the ASL pathology.

This mechanism can also be extended to other inflammatory disease and the connection of TEs upregulation with inflammatory signaling could be targeted for specific therapies. Systemic lupus erythematosus (SLE) and Sjögren’s syndrome are autoimmune diseases in which the overexpression of Alu elements enhances the inflammatory autoimmune response. It has been described that, in these syndromes, autoantibodies are produced to target the RNA binding protein Ro60. Ro60 is able to bind an RNA motif derived from endogenous Alu retroelements, and its disfunction results in enhanced expression of Alu RNAs that then promotes *IFN*- type I regulated genes upregulation, feeding the inflammatory phenotype of the diseases [76]. Alu expression was discovered as directly proportional with interferon signature metric (ISM) level of SLE patients. This study attributes the pathogenicity of anti-Ro60 autoantibodies and type I interferon in SLE and Sjögren’s syndrome to Alu retroelements. However, further studies are required to evaluate the potential of Alu and other Ro60-associated RNAs to activate the IFN response in health and disease [77].

Aicardi-Goutières syndrome (AGS) is an inflammatory disorder, most typically affecting brain, characterized by the dysregulation of type I IFN levels due to mutation in several factors like TREX1 (DNA sensing pathways 3′repair exonuclease) that plays a critical role in restricting the amount of endogenous DNA in the cytosol, and ADAR1 (adenosine deaminase), acting on RNA sensing pathway [78]. Thomas et al. demonstrated in AGS that *TREX1* mutation permits extranuclear accumulation of L1 reverse transcribed cDNA (ssDNA), that triggers inflammation by IFN type I secretion, identifying these molecules as source of neuroinflammation [79]. L1 accumulation has been shown to induce neurotoxicity in neurons and astrocytes; the use of reverse transcriptase inhibitors in AGS neurons and organoids model rescued this phenotype, suggesting the potential use of these inhibitors in treating AGS and related disorders. Similarly, the mutation in *ADAR1* improves cytosolic L1 ssRNA levels increasing the IFN production [65]. Moreover, a recent study performed in a cellular model of senescence and inflammation proposed that L1 become transcriptionally de-repressed in late senescence, the RNA is retrotranscribed and the derived cDNA (ssDNA) activates IFN-I response; this work revealed a contribution of transposable elements to the senescence-associated inflammatory secretory phenotype, suggesting that L1 reverse transcriptase inhibition could be a therapeutic target for the age-associated disorders [80].

Overall, this evidence suggests that TEs are fine regulators of the immune response and that, in particular, their deregulation is associated with pro-inflammatory and autoimmune phenotypes, suggesting that TEs, being able to impose an aberrant activation of the immune response, could represent important yet under-investigated players in complex and multifactorial inflammatory diseases.

### 2.3. TEs RNAs are Novel Players in Cancer Immunity

The tumor microenvironment is represented by malignant, stromal and immune cells, the latter actively involved in tumor fight. Indeed, at first, the immune system is able to destroy and kill cancer cells, but with tumor progression, cancer develops a specific transcriptional program that escapes or attenuates the immune surveillance, rendering the immune cells dysfunctional [81,82,83,84,85]. The immune system regulation acquired a great relevance in cancer therapy and the immunotherapy based on the principle that the immune cells infiltrating the tumor can be reactivated in order to unleash antitumor response [86].

However, it is worth to notice that the tumor, by the above-mentioned mechanisms, can in principle stimulate the immune response against cancer by promoting TEs expression. TEs are under control of DNA methylation and methylation of the lysine 9 or 27 of histone H3 that repress their transcription and block the TEs mediated immunostimulatory activity [87] (Figure 2C). It has been demonstrated that DNA methyltransferases activity, by inhibiting the expression of TEs, permits the evasion of immune surveillance in cancer [63,88,89]. Conversely, the inhibition of DNA methylation reactivates TEs and promotes the innate and adaptive immune response against cancer cells [63,88,89,90] (Figure 2C). In 2015, Chiappinelli et al. demonstrated that, in ovarian cancer, the use of DNA methyltransferase inhibitors (DNMTis) promotes ERVs expression, whose cytosolic dsRNA is recognized by RIG-I and MDA5, further inducing IFN-β production and triggering the immune response [63] (Figure 2C). Similarly, it has been proven that the colorectal cancer initiating cells (CICs, promoting tumor relapse and affecting patient survival) treated with low doses of DNMTis experimentally accumulate cytosolic ERVs dsRNA. ERVs dsRNA are recognized by MDA5 receptor, support the downstream activation of IRFs and the upregulation of interferon-responsive genes. This reduces the proliferation of CICs displaying anti-cancer effects [89] (Figure 2C). In agreement, Goel and colleagues found that inhibitors of cyclin-dependent kinase 4/6 (CDK4/6) increase ERVs expression, to promote the cytoplasmic accretion of ERV dsRNA and increase IFN type III secretion [88] (Figure 2C). Further, these inhibitors suppress CD4+ T regulatory cells proliferation, increase tumor immunogenicity and promote the cytotoxic response by T cells enhancing tumor cells clearance [88]. In acute myeloid leukemia (AML), Cuellar and colleagues [90] demonstrated that the silencing of H3K9 methyltransferase SETDB1 leads to the overexpression of different TEs, promoting IFN antiviral response through dsRNA-sensing pathway [90] (Figure 2C). Other epigenetic histone marks as the repressive H3K27me3 are involved similarly in the regulation of cancer—promoted immune response. A novel subclass of ERVs, the SPARCS, are silenced by EZH2. The inhibition of EZH2, from one side promotes SPARCS expression that in turn activates the dsRNA-sensing pathway and the IFN response, and also on the others induces old MHC class I upregulation and neoantigens presentation, with an overall stimulation of the tumor T cells infiltration and immune activation, suggesting a possible role for TEs in cancer therapy such as adjuvant molecules used in combination with immunotherapies [91].

Very recently, Smith et al. [92] proposed that, besides the ERVs mediated dsRNA promotion of the innate immune response involving the RIG-I pathways, novel retroviral epitopes expressed by the tumor cells drive T and B cell responses, promoting the adaptive immune activation. Importantly, Smith et al. demonstrated that ERVs expression could be used as a prognostic biomarker for outcome of immunotherapies [92].

These studies corroborate the idea that in TEs could reside novel, regulatory molecules able to modulate tumor immunogenicity and anti-tumor immune response, contouring TEs as novel molecules to be investigated in cancer immunotherapy.

## 3. TEs Transcriptional Landscape in Cancer Tissue

### 3.1. The Expression of TEs is Widely Dysregulated in Cancer Tissue

Genetic alterations are recognized as major causes of neoplasia, being able to further promote transcriptional alteration in cancer [93]. In such complexity, TEs are widely dysregulated during cancer development [94] and in many different tumor types [95]. The expression of TEs can promote retrotransposition and the human genome has evolved mechanisms, at both the transcriptional and post-transcriptional level, to avoid detrimental effects on the host genome [24]. While physiologically DNA repetitive sequences and transposons are highly methylated and repressed [96], human cancers can display hypomethylation specifically in definite genomic regions, as in certain classes of TEs promoters, increasing their expression and retrotransposition [95,97]. Other epigenetic modifications can be altered in cancer promoting TEs expression: glioma patients with pervasive H3K27 acetylation display ERV overexpression [98], and as mentioned above, mutation in the epigenetic modifiers for H3K9me3 and H3K27me3 could induce the expression of different classes of TEs [90,91].

Interestingly, the majority of the actively expressed TEs in cancer are those evolutionary youngest such as piggyBac, L1HS, HERVK, and HERVH [95]. Being often full-length elements, these retain intact regulatory regions and behave as active binding sites for transcription factors, possibly further contributing to the transcriptional alteration of cancer tissues [99]. Importantly, it is emerging that TEs expression and dynamics are cancer-tissue specific, similar to what it has been demonstrated for normal tissues [95,100]. Kong et al. [95] have analyzed 7345 TCGA RNA-seq deriving from 25 cancer types in comparison with their normal adjacent tissue counterpart, retrieving an increased TEs expression in certain tumors (stomach, bladder, liver, and head and neck) and a reduced one in others (thyroid, breast, kidney chromophobe, and lung adenocarcinoma). Further, TE subfamilies show a specific expression across all the tumor types. Out of the 19,057 TE subfamilies, 587 display a different expression in at least one TCGA cancer, 80% of which were overexpressed and belonged to LTR and LINE classes. The overexpression of LTRs, particularly HERV, has been found in different epithelial tumor types, including colorectal cancer (CRC) [101], melanoma [102,103], renal cell carcinoma [104], pancreatic adenocarcinoma [105], glioma [98], as well as breast [106] and ovarian cancer [107]. L1 has been reported as aberrantly expressed in many cancers (breast, head and neck, lung [108]), and CRC [109]. Interestingly, in CRC patients, L1 expression depends on DNA damage repair ability, where Microsatellite Instable (MSI, mutated in the DNA repair machinery) cancers show a lower expression of L1 in respect to Microsatellite Stable (MSS, not mutated), reflecting different DNA methylation levels [109].

**Figure 3:**
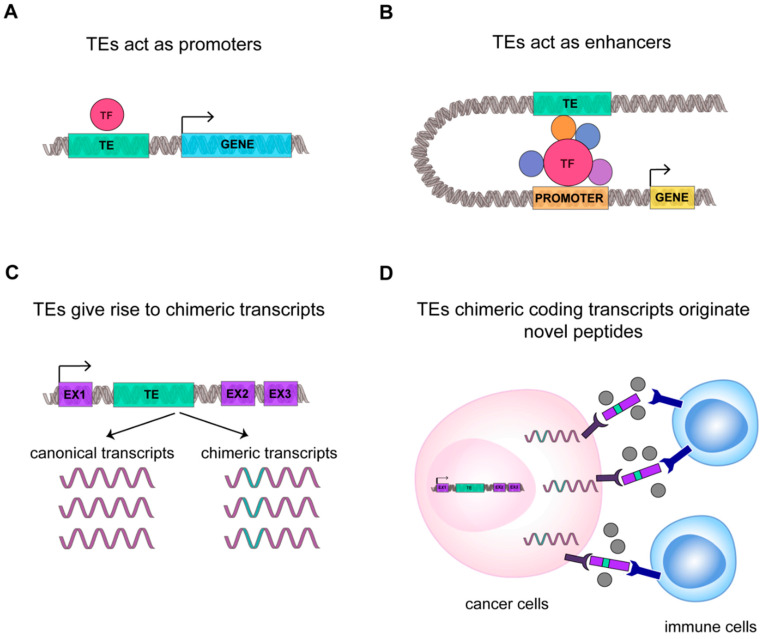
TEs transcriptome contributes to cancer transcriptional fingerprint. A schematic representation of new function mediated by TEs in cancer: (**A**) TE (in green) can act as promoter sequence or (**B**) enhancer sequence. Transcription Factor and cofactors (TF) are highlighted in red and violet. (**C**) TEs can generate new chimeric transcripts, (**D**) giving origin to new oncogene transcripts and peptides that can be recognized by immune system as not-self, improving cancer immunogenicity.

TEs deregulation in cancer can contribute to TEs use as promoters [110]. The promoter activation can further lead to the expression of the genes surrounding TEs, which may contribute with TEs to tumorigenesis in a synergistic or cooperative manner [110] (Figure 3A). The specific mechanism by which TEs are able to promote the tumorigenesis and tumor progression remain mostly unknown [111]. Within this frame, a possibly critical function for L1 expression has been suggested in early phases of cancer formation, setting up the gene expression profiles favorable to tumor development [112]. Indeed, in CRC a correlation has been described between disease stage and L1 hypomethylation and expression [113]. This finding has also been confirmed for ERV in the endometrial carcinoma growth [114], suggesting a correlation between TEs and the establishment of a cancer specific program.

Collectively, these findings suggest that TEs deregulation can be specifically involved in the establishment of cancer-specific transcriptional programs, suggesting that transposons can be co-opted for cancer fitness and survival, and that these elements could be used in defining novel molecular classifications.

### 3.2. TEs RNAs Improve Cancer Specific Transcriptional Complexity and Plasticity

TEs can contribute to cancer specific functions acting at different level of transcriptional regulation. It has been reported that TEs, dispersed across the human genome, represent a huge reservoir of gene regulatory modules, both promoters and enhancers [110,115,116] and that transposons can mediate the genesis of new transcripts [116,117,118], possibly contributing to the translation of new cancer-specific peptides [95].

Cancer-specific co-option of transposable elements takes the name of onco-exaptation, a term coined by Babaian et al., to describe the mechanism by which epigenetically repressed TEs have been harnessed to promote human oncogenesis (Figure 3) [115]. Babaian et al., analyzed RNA-seq datasets from nine Hodgkin lymphomas (HL), finding that proinflammatory transcription factor IRF5 was upregulated in HL-derived cell lines due to the transcriptional activation of the retroviral LOR1a LTR as regulatory enhancer (Figure 3B) [115]. Similarly, in 2010, Wolff et al. found in bladder cancer that the demethylation of a specific L1 promoter induces activation of an alternative transcript of the *MET* gene, that codifies for a permanently active MET protein, a tyrosine kinase receptor that promotes tumor growth (Figure 3A) [110]. For ALK-negative anaplastic large cell lymphoma it was demonstrated that the reactivation of LTRs causes the expression of a novel isoforms of the oncogene *ERBB4*, a type I receptor tyrosine kinase [119]. In agreement, Jang et al. characterized the activation of TE-derived cryptic promoter in 7769 tumors and 625 normal tissues, found TE-derived oncogene transcripts in 15 cancer types, and proposed that this mechanism contributes to oncogene activation in about half of all malignant diseases [116]. This result suggests that transposons can contribute to the genesis of new chimeric transcripts (Figure 3C). Likewise, the activation of L1 promoters, due to the loss of DNA methylation, can promote the transcription of nearby regions, generating cancer-specific L1 chimeric transcripts [117]. The L1-chimeric transcript *LCT13*, transcribed from the L1 antisense promoter, behaves as ncRNA silencing the tumor suppressor TFPI-2 and promoting cancer progression [118]. In diffuse large B-cell lymphomas 98 TE-gene chimeric transcripts have been found and the expression of *LTR2-FABP7* chimeric transcript was suggested to code for a novel protein able to positively influence diffuse large B-cell lymphoma cell proliferation [120]. Notably, in different cancers, 83 unique peptides derived from TEs chimeric transcripts have been identified, among which 39 were common to different tumor tissues [95]. We can hypothesize that these new peptides originating from TEs chimeric transcripts could increase the number of cancer-associated neoantigens, possibly rendering tumor more susceptible to immunotherapy (Figure 3D) [95].

Collectively, these data suggest that TEs can be deeply involved in orchestrating cancer type–specific regulatory networks, increasing cancer transcriptional complexity and plasticity, and further promoting tumor adaptability and fitness.

### 3.3. TEs Regulate Cancer Tumorigenicity and Progression

It is accepted that TE mediated retrotransposition can act at the genomic level, promoting genome instability and cancer progression [16,121,122]. However, very little is known regarding the functional correlation between TEs expression and cancer establishment and progression.

In pancreatic cancer cell lines, it has been demonstrated that L1 and HERVK silencing reduce the tumorigenicity of the cells inoculated in nude mice [105]. Aschacher et al. [123] show evidence that downregulation of L1 in different cancer cell lines induces telomere shortening and consequently slower spheroid cancer cell development, promoting a G2 phase cell cycle arrest and suggesting L1 involvement in cancer cell proliferation. In hepatocellular carcinoma it has been proposed that L1 RNAs can have a function in cellular transformation, through a splicing-mediated regulation of the protooncogene G antigen 6 (GAGE6). This suggests that endogenous L1 RNA may display regulatory functions in the process of tumorigenesis [124]. Finally, functional analyses in leukemic stem cells revealed a specific contribution of TEs classes to inflammation, the expression of SINE and LTR positively correlating with inflammation, and L1 anticorrelating [125]. The authors add clinical values to this discovery, hypothesizing that TEs can be used and targeted to modulate the immune response with tumor microenvironment.

Other possible mechanisms by which TEs can promote cancer progression are described for HERVs elements, that could initiate the transcription of ncRNA and lncRNAs with oncogenic properties [126]. The 5′ end of LTR7 element induces the expression of the pro-oncogenic lncRNA ROR [45] in different cancer types [127] while a HERVK 11 ncRNA binds to polypyrimidine tract-binding protein-associated splicing factor, inhibiting the repression of proto-oncogene transcription and consequently leading to cell transformation and tumorigenesis [128,129]. However, HERVs RNAs can display also tumor suppressive function, as for the antisense transcript of ERV-9 LTR that in normal cells physically binds transcription factors involved in cell proliferation, and that is downregulated in malignant cells sustaining uncontrolled cancer growth [130,131].

An additional evolutionary way by which TEs RNAs drive tumor progression are the microvesicles, small lipid bilayer extracellular vesicles released from cells that could contain RNAs, DNA fragments, peptides, and lipids [132]. Retroviral-like microvesicles have been found in the plasma of cancer patients [133,134,135]. In particular, in vitro studies showed that tumor-derived microvesicles are enriched in HERV, L1, and Alu DNA and RNAs, that could be transmitted to other cancer and normal cells thanks to macrovesicles fusion with the cellular membranes [136]. Microvesicles are recently shown to drive cancer growth and proliferation and to regulate near or distant healthy cells within tumor microenvironment [126].

Combined, these data strongly suggest that TEs can be specifically involved in promoting tumorigenesis and cancer progression in a wide set of cancer types acting via different mechanisms, that, beside the more obvious genomic effects of the retrotransposition, surprisingly also involve the RNA counterpart of these elements.

## 4. Next-Generation Sequencing (NGS) Approaches for the Analysis of TEs

### 4.1. Dealing with Ambiguity in RNA-Seq Reads Alignment: A Challenge to Resolve TEs Expression Quantification

**Figure 4:**
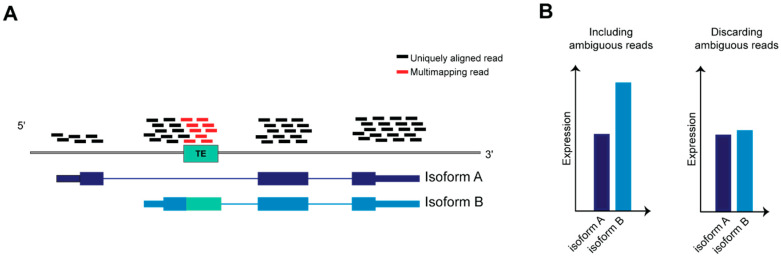
Ambiguous reads in transcript quantification. (**A**) Schematic representation of RNA-seq reads aligned on a gene on the reference genome, the gene is transcribed in two transcript isoforms, A and B. (**B**) Isoform B is twice more abundant than A; however, if ambiguous reads are discarded from reads count, the difference between A and B will be negligible after normalizing read counts against transcript length.

TEs have been co-opted in different biological scenarios representing novel molecules able to regulate the tissue specific transcriptional networks that establish in physiological and pathological context. The advent of evolving NGS technologies, the formation of international consortia that produced a multitude of datasets and developed bioinformatic tools have been indispensable for realizing how broad is TEs involvement in mammalian biology, and depicting precise function for certain classes, superfamilies and subfamilies of TEs in a given spatiotemporal frame. However, in order to precisely define the contribution of a given TEs locus to the regulatory networks of specific genes, it is important to identify and characterize TEs at the genomic instance resolution. A systematic and unambiguous analysis of TEs (that are repeated in several highly homologous interspersed genomic loci) at the genomic instance level or within genes containing TEs using RNA-seq is a non-trivial task (Figure 4), due to the limitations of mapping algorithms, which do not allow the assignment of multi-mapping reads to a precise genomic locus [137].

Here, we provide a comprehensive overview of the technological progresses in NGS technologies and computational methods, from the sequencing design (e.g., read length and pairing) to the development of specific tools for the downstream analysis of TEs annotation and expression. Also provided is an outline on the contribution of the knowledge that we have acquired and previously summarized on TE functions in genome biology.

Some precautions in the library preparation can help mitigating the amount of multi-mapping TE-derived reads, such as using a paired-end layout and a longer read length to make more likely that the read will contain a unique genomic sequence that can be mapped. However, long repeat instances, such as LTR and LINE retrotransposons, can span from hundreds to thousands of nucleotides, challenging an unambiguous identification via the current, state-of-the-art RNA-seq protocols. Some of the longest TEs harbor an intact promoter and ORF sequences, and are therefore able to be transcribed and to retrotranspose under conditions that cause the removal of their repression, such as hypomethylation in cancer (see Section 3.1). Therefore, being able to resolve the quantification of these TEs can be crucial to properly study the contribution of transposons in such pathological conditions.

Since the early years of the NGS era, multi-reads have been handled in different ways, each with its own advantages and drawbacks: i) ignoring multi-reads by selecting unique alignments only. This option may lead to underestimating the expression levels of TEs and their derivates, as well as the overall expression level of a sample, but assigns reads with the highest confidence; ii) reporting the best alignment for each multi-mapping read based on the alignment quality score calculated by the mapping algorithm. Here, the results may vary based on how mismatches and gaps between the reads and the reference genome are weighted, making it difficult to provide the exact genomic location with high confidence; iii) keeping multi-reads, counting them once for each mapped feature. This prevents discarding potentially relevant loci from the downstream analysis. However, genomic features characterized by a high number of multi-reads, as well as the total library size, will be overestimated.

To avoid discarding relevant biological signals from multi-mapping or ambiguous reads, multi-mapping reads should be either assigned to a unique genomic feature or re-distributed across the multi-mapped regions. To accomplish the assignation to a unique genomic feature, available methods implement algorithms to assign, according to different criteria (see below), the genomic feature that is the source of transcription for those reads. Whenever this is not possible, the reads can be assigned computing a probability, they will be proportionally re-distributed across the mapped genomic features according to how likely they are to be the source of transcription (often based on the level of transcription of the genomic features, see below). This approach offers a more precise estimation of expression and reads coverage across genes, and some of the methods implementing it are discussed below.

In 2008, Mortazavi et al. [138] depicted one of the earliest efforts in this direction, in which multi-reads are recovered by distributing them across the aligned genes, proportionally to the amount of unique alignments on a given gene. This method resulted in an increase of expression levels estimates by more than 30% compared to discarding multi reads, for several mouse genes.

The importance to use multi-reads in gene expression profiling of cancer, has been more recently considered by Robert and Watson [139] with a survey on 12 common methods for gene-level expression quantification from RNA-seq data. The expression levels of hundreds of genes are underestimated by one or more of those methods; interestingly, many of these genes are implicated in human diseases. The quantification of such genes is proposed via multi-map groups (MMGs) of genes that multi-reads map to, and by this approach, MMGs are differentially expressed between normal and lung tumour mouse cells, while the methods based on unique counts failed to produce this result [139]. By avoiding quantifying the expression of individual ambiguous genes, Robert and Watson could retrieve important data that otherwise would have been missed, but, on the other hand, the information on the transcripts is not considered in the analysis. This technical gap was filled by the multi-mapper resolution tool (MMR), developed by Kahles et al. in 2016 [140]. In contrast to the previous methods, MMR returns an expression estimate for each individual gene or transcript, and it does not proportionally distribute multi-reads across the aligned features. Rather, MMR assumes that the reads coverage should be uniform within a local region, thus selecting the alignment that leads to the smoothest coverage signal across a window of a fixed length.

Recently, pseudo-alignment algorithms emerged as an alternative to aligning RNA-seq reads to a reference genome, by directly inferring the transcript from which the read originates [141,142]. The ambiguity of highly overlapping transcripts in the human genome is circumvented by probabilistically distributing the reads count across a given transcriptome, avoiding the generation of multi-reads in the first place. Tools based on pseudo-alignment have become valuable in transcriptomics, providing a fast and reliable method for transcript-level quantification.

Besides RNA-seq, several NGS methods are designed to meet specific needs in transcriptome analysis. Among these, cap analysis of gene expression (CAGE) is an high-throughput technology for sequencing the 5′ end of transcripts into short reads (tags) [143]. CAGE has been proven valuable for the discovery of novel transcription start sites (TSS) of either novel genes or alternative transcript isoforms of known genes [144]. Faulkner et al. [145] showed a method to recover short multi-reads produced by tag-based NGS technologies such as CAGE, in which a score is given to tag-TSS associations according to the amount of individual tags associated to the same TSS; multi-mapping tags are proportionally assigned to the mapped TSS according to the calculated scores. With this method, it has been demonstrated that up to 30% of transcripts initiate from within TEs [36], and that some of them are associated with enhancer regions in stem cells, regulating their pluripotency [46].

Therefore, rescuing multi-mapping CAGE tags, or multi-reads in other NGS technologies complementary to RNA-seq, has been fundamental in clarification of the extent to which TEs influence the transcriptional output of mammalian cells in both physiological and pathological contexts.

### 4.2. Current Computational Methods for TEs Transcriptome Analysis

General-purpose computational methods, such as the aforementioned ones, help with the recovering of ambiguous reads for their inclusion in downstream analyses, including those originating from TEs. However, some contexts of analysis require complementary specialized tools designed for TEs to survey the overall contribution of the various TE categories to the transcriptional output of a certain tissue, or to be able to properly distribute the RNA-seq signal among active TE instances and TEs expressed as part of other transcripts.

Several TE-centric tools have been developed to (i) identify and quantify expressed TEs from transcriptomic datasets that can be classified based on their capability of quantifying TE expression at the subfamily level (counting a subfamily as an individual entity) or at genomic instance level (to quantify the expression of individual elements), and (ii) discern TEs that are actively transcribed as individual transcriptional units from those that are co-expressed within other transcripts (Table 1).

Criscione et al. [146] published RepEnrich in 2014. They rescued most multi-reads by assigning them proportionally to the subfamilies on which they align, and showed that many TEs subfamilies are expressed in a tissue-specific manner, and significantly enriched in cancer [148]. Recently, Jung et al. [156] used TEtranscripts to improve the expression estimate of L1Hs in cancer, potentially active in the human genome. By quantifying L1Hs somatic insertions and their overall expression in whole-genome and RNA sequencing data from matched TCGA gastrointestinal cancer samples, they found that L1 insertions count and expression are significantly higher in cancer tissues compared to normal, and that L1 insertions causes abnormal mRNA splicing and gene expression [156].

TEtranscripts does not discern potentially autonomously transcribed TEs from pervasively transcribed ones. To do that, Navarro et al. [155] recently released TeXP method that removes the noise due to pervasive transcription from the RNA-seq signal mapping on evolutionarily young subfamilies. [155]. They applied this method in several RNA-seq datasets from cancer and healthy human cell lines and tissues, and found a greater amount of autonomous transcription for transposons in the human germline and in tumor cell lines.

A different approach to quantify the expression of TEs at class, superfamily or subfamily level is to align RNA-seq reads on a custom transcriptome of TEs sequences, rather than a reference genome. TEtools [153] is a pipeline that works in this way, enabling the analysis of a TE transcriptome by providing the sequences of TE instances and computing a class-superfamily-subfamily level count and a differential expression analysis. A recent work by Cebrià-Costa et al. used TEtools to perform a differential expression analysis of TEs in an epigenetic study on the function of histone 3 lysine 4 oxidation by LOXL2 in breast cancer cells, and to rule out the possibility that the overexpression of TEs were responsible for DNA damage response in LOXL2 KD cells [157].

As aforementioned, pseudo-alignment can quantify transcripts including both unique and ambiguous reads, avoiding the generation of multi-reads. Recently, TE-centric pipelines based on pseudo-alignment have been released as SalmonTE [149] and REdiscoverTE [95], that both leverage on Salmon’s pseudo-alignment algorithm. Kong et al. illustrate REdiscoverTE using over five million genomic repetitive elements annotated by RepeatMasker [158] together with cDNA transcript sequences as well as the sequences of introns containing repetitive elements. They show that including all genomic repeats instances in the reference transcriptome allows taking in account the sequence diversity within TE subfamilies. This includes eventual genomic TE loci that significantly deviate from the Repbase consensus sequence, and results in a more accurate quantification of TE hierarchies. Further, the inclusion of intronic sequences containing repetitive elements allows mapping reads on TEs transcribed within unannotated alternative exons or retained introns. By applying this pipeline on 7750 TCGA cancer samples, Kong and colleagues [95] described the TE expression landscape in cancer, differentiated between the TEs co-expressed within host genes and intergenic TEs, and found the latter more expressed and more correlated with DNA demethylation, DNA damage and immune response in cancer [95].

Measuring the expression enrichment of TEs in RNA-seq data when comparing different cell types, developmental stages or pathological conditions can provide important evidences on the regulatory network in which TEs are involved. However, to deeply investigate TEs involvement in a specific mechanism or phenotype, it is crucial to study TEs expression at the individual genomic instance resolution. Indeed, for example, a different function would be expected for evolutionarily old TEs in respect to the youngest ones that own a promoter and are able to retrotranspose in the genome. For this purpose, Yang et al. published SQuIRE in 2019, the first bioinformatics tool designed for locus-specific quantification of interspersed repeats [150], based on the spliced alignment of a reference genome of RNA-seq data. By applying this method, they show a differential expression of individual TE instances across different tissues of healthy mouse, as well as of TEs differentially expressed in a *D. Melanogaster* model of amyotrophic lateral sclerosis, highlighting the structure of the transcripts containing such TEs, that would not have been possible without a locus-level resolution.

Besides SQuIRE, other tools reports the expression estimates of TEs at genomic instance level [146,151] by L1EM tool [146] that has been developed to quantify the expression of autonomously transcribed L1 elements at locus level. As reported by L1EM analysis, full-length L1 loci of the L1Hs subfamily are highly expressed in stem and cancer cells, while being less expressed in differentiated tissue samples.

Bioinformatics analyses in TE-centric studies may not be limited to the expression of TEs instances. As we reviewed, TEs influence the transcription of coding and non-coding RNAs in several ways [36]. Jang et al. characterized the landscape of TE onco-exaptation across RNA-seq data from TCGA tumors and normal samples, which they reanalyzed using a pipeline for transcript assembly and integrated with data from the FANTOM5 consortium for the annotation of TE-derived transcription start sites [116]. This analysis revealed the prevalence of TE usage as novel regulatory sequences in cancer and its importance for oncogene activation and tumorigenesis. In this context, a recent tool, LIONS [147], is specifically designed to detect and quantify transcripts initiated from within TEs. This tool is able to estimate expression levels of both TEs and exons, and to compute a specific metric to discern TE-initiation from TE-exonization events based on read coverage. Finally, if more than one experimental group is being processed, LIONS performs a differential analysis between them.

Alternative approaches for an accurate quantification of TEs expression could also use data generated by new technologies, although less available than RNA-seq. For example, Deininger et al. developed a pipeline based on RNA-seq and 5′ RACE coupled with PACbio sequencing of 1200 base pair-long reads to estimate the expression of L1 RNAs expressed as independent transcriptional units [159]. In particular, they show that a large part of the total expression of full-length L1 elements derives by the transcription of a relatively small number of L1 loci. Indeed, this method anticipates the potential of long read sequencing in identifying the TEs contributing to the majority of expression and new insertions in several cancer conditions. Indeed, recent advancements on long-read sequencing, that obtain and map tens of thousands of base-pair long reads, should allow to identify the TEs expressed and contributing to new insertions in cancer conditions, and may signal a new era for the analysis of TEs in transcription regulation, other than for genomics as a whole [160,161].

Despite the limitations of NGS technologies for studying interspersed repetitive elements, recent efforts in bioinformatic research have undoubtedly reached the goal of increasing the level of confidence by which the expression levels of such elements are estimated, and enabled the discovery of several transcriptional regulatory networks in which they are involved in physiological and pathological conditions. Nonetheless, further efforts are still required to improve bioinformatic practices and increase the awareness of the biological relevance of the once called “junk DNA”.

## 5. Conclusions

In the current review, we summarized the latest findings on TEs, highlighting that, beyond their ability of being “jumping elements”, they contribute to the establishment of a vast regulatory network that, controlling genome plasticity, magnifies the cell type specific transcriptional complexity, both in health and diseases.

Although TEs are finely transcriptionally regulated in order to avoid the negative effects of their transposition, TEs activation physiologically takes part within the concerted spatio-temporal establishment of the cellular transcriptional programs. Indeed, we find that TEs can mediate multi-layered regulatory functions in cellular identity establishment in embryogenesis and development (see subheading 1) and that using the host signaling pathways, TEs are key players in the regulation of adult tissue plasticity and adaptation to environment, as occurs in the innate and adaptive immune response (see Section 2). Further, we also highlighted how TEs can represent evolutionary instruments that create novel functions that can be positively selected to promote cancer fitness and tumorigenesis (see Section 3). Importantly, we showed that many of these findings have been achieved through the employment of NGS sequencing technologies and bioinformatic tools that are in continuous development and improvement to overcome the limitations of TEs study that render their unambiguous annotation and analysis still puzzling (see Section 4).

Concluding, TE mediated regulatory networks represent a prolific source of still hidden players that could explain complex phenomena such as the establishment and progression of multifactorial diseases. Transposons represent a new window for novel therapeutic opportunities and for deriving targetable molecules for personalized based therapies. For these purposes, it is necessary to develop computational and experimental methods to identify and characterize more systematically TEs at their genomic instances, in order to improve our knowledge about their implications in genome plasticity and functions in health and disease.

## Figures and Tables

**Table 1:** Computational tools and pipelines for transposable elements (TEs) transcriptome analysis.

Name	Resolution	TE Specificity	Detection of Active Transcription	Method Description	Reference
REdiscoverTE	Subfamily	All	Yes (Intergenic TEs are classified as autonomously transcribed)	Pseudo-alignment on a transcriptome of cDNA and individual genomic loci.	[95]
L1EM	Locus-level	LINE1	Yes	Categorizes L1 loci by the presence of promoter and polyA tail; EM-based quantification.	[146]
LIONS	Locus-level	TEs initiating transcripts	No	Identify and quantify TE-initiated transcripts based on read coverage on *de-novo* reconstructed exons and around TEs.	[147]
RepEnrich	Subfamily	All	No	Non-spliced alignment on a pseudo-genome of repeats sequences.	[148]
SalmonTE	Subfamily	All	No	Pseudo-alignment on TE consensus sequences.	[149]
SQuIRE	Locus-level	All	No	Spliced alignment followed by EM-based locus-level quantification.	[150]
TEcandidates	Locus-level	All	No	Alignment of *de novo* assembled contigs of TE-derived reads to the reference genome.	[151]
Telescope	Locus-level	All	No	Reassignment of multi-reads to the most probable source of transcript.	[152]
TEtools	Subfamily	All	No	Reference-free alignment on a provided set of TE sequences.	[153]
TEtranscripts	Subfamily	All	No	EM-based re-distribution of pre-aligned multi-reads.	[154]
TeXP	Subfamily	All	Yes	Removes *noise* derived from non-autonomous transcription of TEs.	[155]

From left to right: name of the software, resolution of expression estimation (e.g., TE (sub)family or locus-level), specificity of the software towards a particular category of TEs, ability of the software to discern autonomous from passive TE transcription, brief description of the method, reference of the associated publication. All the software listed in this table, including the source code, are freely available.

## Abbreviations

LINE	Long interspersed element
L1	Long interspersed element 1
SINE	Short interspersed element
LTR	Long terminal repeat
TE	Transposable element
ERV	Endogenous retrovirus
NGS	Next generation sequence
IFN	Interferon
NK	Natural killer
DC	Dendritic cell
NF-κB	Nuclear factor kappa B
AP-1	Activator protein-1
IRF	Interferon response factor
PRR	Pattern recognition receptor
PAMP	Pathogens associated molecular patterns
RLR	retinoic acid-inducible gene I-like receptors
RIG-I	Retinoic acid inducible gene-I
MDA5	Melanoma differentiation-associated gene 5
TLR	Toll like receptor
cGAS	GMP-AMP synthase
AID	Activation-induced cytidine deaminase
ss	Single strand
ds	Double strand
lnc	Long non coding
CRC	Colon rectal cancer
AGS	Aicardi-Goutières Syndrome
TREX1	DNA sensing pathways 3′repair exonuclease
ADAR1	Adenosine deaminase acting on RNA
DNMTi	DNA methyltransferase inhibitors
AML	Acute myeloid leukemia
CDK4/6	Cyclin dependent kinase 4/6
SLE	Systemic lupus erythematosus
ALS	Amyothropic latelar sclerosis
CIC	Cancer initiating cell
MSI	Microsatellite instable
MSS	Microsatellite stable
HL	Hodgkin Lymphoma
GAGE6	protooncogene G antigen 6
